# 1,25-dihydroxyvitamin D in the elderly population: Comparison of liquid chromatography tandem mass spectrometry and CLIA immunoassay (LIAISON®XL) methods

**DOI:** 10.1016/j.plabm.2025.e00474

**Published:** 2025-05-08

**Authors:** Dorota Leszczyńska, Alicja Szatko, Magdalena Ostrowska, Magdalena Zgliczyńska, Konrad Kowalski, Wojciech Zgliczyński, Piotr Glinicki

**Affiliations:** a Department of Endocrinology, Centre of Postgraduate Medical Education, Warsaw, Poland; b EndoLab Laboratory, Centre of Postgraduate Medical Education, Warsaw, Poland; c Doctoral School of Translational Medicine, Centre of Postgraduate Medical Education, Warsaw, Poland; d Department of Obstetrics, Perinatology and Neonatology, Centre of Postgraduate Medical Education, Warsaw, Poland; e Masdiag Laboratory, Warsaw, Poland

**Keywords:** 1,25-Dihydroxyvitamin D, 1,25(OH)_2_D, Vitamin D, LC-MS/MS, Immunoassay, CLIA, LIAISON®XL, Hypercalceamia, Elderly population, Metabolomics

## Abstract

**Background:**

1α,25-dihydroxyvitamin D is the biologically active form of vitamin D_3_ (cholecalciferol) and vitamin D_2_ (ergocalciferol). The determination of 1,25(OH)_2_D is clinically relevant in the diagnostics of vitamin D metabolism disorders, PTH-independent hypercalceamia and hypophosphatemic syndromes. The quantitative assessment of 1,25(OH)_2_D may be a challenge since it circulates in picomolar concentrations in the blood.

**Aims of the study:**

Comparison of two methods: chemiluminescent immunoassay (CLIA, LIAISON®XL) and Liquid Chromatography Tandem Mass Spectrometry (LC-MS/MS) for determinations of 1,25(OH)_2_D concentrations in elderly populations. The secondary aim was to assess correlation between 1,25(OH)_2_D for CLIA_LIAISON®XL_ and LC-MS/MS methods and selected factors (vitamin D metabolites, calcium, albumin, PTH, creatinine concentrations in serum and age).

**Materials and methods:**

The study was conducted on 54 patients aged from 60 to 96 at the Bielański Hospital in Warsaw, Poland. The determination of 1,25(OH)_2_D using CLIA_LIAISON®XL_ and LC-MS/MS methods was performed.

**Results:**

Both methods (CLIA_LIAISON®XL_ immunoassay and LC-MS/MS technique) were strongly positively correlated (r = 0.86; p < 0.001). In the LC-MS/MS technique, concentration of 1,25(OH)_2_D was significantly higher compared to the CLIA_LIAISON®XL_ immunoassay. The regression equation revealed method interchangeability. Concentration of 1,25(OH)_2_D was significantly correlated with various basic biochemical parameters (albumin and calcium levels) for both methods.

**Conclusions:**

In our study, measurement of 1,25(OH)_2_D using CLIA_LIAISON®XL_ was not inferior to LC-MS/MS measurement. The assessment of 1,25(OH)_2_D using CLIA_LIAISON®XL_, characterized by short turnaround time, low costs and high accuracy, may be an optimal choice for elderly patients who often require prompt diagnosis and treatment.

## 1 Introduction

The multidirectional effects of vitamin D, which extend far beyond its classical skeletal function, has significantly heightened interest in measuring vitamin D metabolites and developing new techniques for its determination. Vitamin D exists in two inactive forms: vitamin D_3_ (cholecalciferol) obtained through skin synthesis or an animal-derived diet and D_2_ (ergocalciferol) acquired from a plant-based diet [1]. The most commonly evaluated metabolite of vitamin D is 25(OH)D (the total of 25(OH)D_2_ and 25(OH)D_3_), the product of 25-hydroxylation of vitamin D_2_ and D_3_ in the liver. 25(OH)D is universally accepted as one of the most reliable biomarkers for assessing the vitamin D status [2]. It is the result of relatively stable expression of the 25-hydroxylase gene (CYP2R1) meaning that the 25(OH)D concentration depends mainly on the availability of its substrate. Another most frequently measured metabolite is 1,25(OH)_2_D, the biologically active metabolite of vitamin D, which is produced through conversion of 25(OH)D by the renal 1α-hydroxylation encoded by CYP27B1 gene (Cytochrome P450 Family 27 Subfamily B Member 1). In addition to kidneys, CYP27B1 is also expressed in several other cell types, however 1,25(OH)_2_D synthesis in these cells is mainly local and does not significantly affect its blood concentration [3]. Unlike 25(OH)D, the measurement of 1,25(OH)_2_D does not reflect the vitamin D status. It is the result of strict regulation of the renal CYP27B1 gene by parathyroid hormone (PTH), serum calcium and phosphate concentrations, and fibroblast growth factor 23 (FGF23), which maintain 1,25(OH)_2_D concentration within the narrow range [3]. Nevertheless, assessing 1,25(OH)_2_D is relevant in diagnosing of vitamin D metabolism disorders, PTH-independent hypercalceamia and hypophosphatemic syndromes. However, the full clinical utility of this measurement however, does not yet appear to be fully recognized and appreciated [4].

1,25(OH)_2_D, as opposed to 25(OH)D, has a shorter half-life (approximately 4–6 h), is more hydrophilic and circulates in the bloodstream in picomolar concentrations [5]. Due to quantitative limitations, the measurement of 1,25(OH)_2_D is regarded as one of the most challenging among all steroid hormones for the analytical biochemist and requires methods with significantly greater sensitivity [6]. In contrast to 25(OH)D, there is currently no generally approved reference method for 1,25(OH)_2_D determination and the variability of measurement between different methods and techniques is considerable [7]. Hypercalcaemia among patients admitted because of emergency reasons is observed up to 2.7 % of cases, more frequently in elderly patients [8]. In the differential diagnosis of hypercalcaemia, evaluation of 1,25(OH)_2_D is very valuable.

The aim of the study was to assess whether the CLIA_LIAISON®XL_ immunoassay provides similar accuracy and reliability in measuring 1,25(OH)_2_D concentrations using as the LC-MS/MS technique in the elderly. Considering its relevant cost, low availability, high throughput and the long time needed for LC-MS/MS analysis, the CLIA_LIAISON®XL_ immunoassay may be an advantageous option, especially in situations requiring prompt diagnosis and treatment. Additionally, considering potential interferences, we assessed the correlation between 1,25(OH)_2_D measured using CLIA_LIAISON®XL_ and the LC-MS/MS technique with selected parameters namely: 25(OH)D_3,_ 25(OH)D_2_, 24,25(OH)_2_D_3,_ 3-epi25(OH)D_3_, serum calcium concentration, albumin, PTH, creatinine, and age.

## 2 Materials and methods

### 2.1 Study population

The study was conducted between December 2022 and June 2023 in secondary care center (the Department of Internal Medicine, Bielański Hospital) in Warsaw, Poland. Patients over 60 years old, who were admitted through the emergency department and had signed written informed consent, were included in the study. The exclusion criterion was kidney insufficiency (creatinine >1.0 mg/dl). The study was approved by the Bioethics Committee of Centre of Postgraduate Medical Education (N° 19/2021).

### 2.2 Sample collections and biochemical tests

Blood samples were collected at a single time point between 8:00 and 10:00 a.m. via venipuncture into Vacuette® tubes (6 mL) with a serum clot activator. After 30 min, the blood was centrifuged at 3500 rpm for 15 min. Serum samples were used for analysis or stored at −86 °C for a maximum of 3 months until the below mentioned measurements. In each sample, the concentration of creatinine, PTH, calcium and albumin was evaluated. Creatinine and calcium were determined using the spectrophotometric method on the Cobas® 6000 analyzer series (Cobas® c501 Roche Diagnostics, Switzerland). Albumin determination was performed using the spectrophotometric method on Cobas® Integra 400 plus analyzer (Roche Diagnostics, Switzerland). PTH concentration was determined using ECLIA (Electrochemiluminescence Immunoassay) immunoassay on the Cobas® 8000 analyzers series (Cobas® e801, Roche Diagnostics, Switzerland).

### 2.3 Determination of 1,25 (OH)_2_D using CLIA immunoassay

Determination of 1,25(OH)_2_D using CLIA was performed on the LIAISON®XL analyzer (DiaSorin, USA). It is a three-step sandwich immunoassay that utilizes a recombinant fusion protein (RFP) and a murine monoclonal antibody (MAB)*.* The LIAISON®XL 1,25(OH)_2_D assay is a fully automated method that requires a 75 μL serum volume and does not require an extraction step. The limit of detection (LOD) is 0.7 pg/ml. The reference range established by manufacturer is 19.9–79.3 pg/mL.

### 2.4 Determination of 1,25 (OH)_2_D by LC-MS/MS technique

#### 2.4.1 Materials

The calibration standards were performed using a calcitriol standard from Dr. Ehrenstorfer (Augsburg, Germany), *d3*-1,25(OH)_2_D_3_ standard, hereafter referred as IS, from IsoSciences (Ambler, PA, USA) and vitamin D-free serum from Golden West Diagnostics (Temecula, CA, USA). Required components to the mobile phases, such as methanol (MeOH), acetonitrile (ACN), ethyl acetate, formic acid (FA), water (H_2_O), were obtained from VWR (Pennsylvania, USA). 4-(4′-dimethylaminophenyl)-1,2,4-triazoline-3,5-dione (DAPTAD), from Masdiag Laboratory (Warsaw, Poland), was used in a derivatization process.

#### 2.4.2 LC-MS/MS instrument parameters and conditions

The analyte separation was conducted using a Cosmocore PBr column (2.6 μm, 100 × 2.1 mm, Nacalai Tesque, Kyoto, Japan) on a Nexera XR HPLC system (Shimadzu, Kyoto, Japan). The detection was carried out in multiple reaction monitoring (MRM) mode on a triple quadrupole tandem mass spectrometer (QTRAP®5500, Sciex, Framingham, MA, USA). The analyte ionization was obtained by the electrospray ionization source (ESI) method in positive ion mode. The following ion source parameters were applied: temperature (TEM) 550 °C, Curtain Gas (CUR) 25 psi, Ion Spray Voltage (IS) 4000 V, Ion Source Gas 1 (GS1) 40 psi, Ion Source Gas 2 (GS2) 70 psi. The MRM transitions for calcitriol were 635.3/357.2 *m*/*z* (quantitative) and 635.3/617 *m*/*z* (qualitative), whereas those for IS were 638.4/360.2 *m*/*z*. The collision energy (CE) was 35V, 25V, and 35V, respectively, as well as the Declustering Potential (DP) of 40V, the Entrance Potential (EP) of 10V, and the Collision Exit Potential (CXP) of 10V.

Mobile phases: phase A (H_2_O) an phase B (ACN), with 0.1 % formic acid, were used with a flow rate of 0.6 ml/min and the temperature 45 °C. Gradient elution was set: 0 min. - 65 % B, 0.6 min. - 80 % B, 8.0 min. - 88 % B, 8.1 min. - 99 % B, 9.4 min. - 99 % B, 9.5 min. - 96 % B. Total duration was 11 min.

#### 2.4.3 Sample preparation

The sample preparation process included precipitation of protein, liquid-liquid extraction (LLE), and derivatization. In this method, 10 μl of IS (10 ng/mL solution in ACN) were added to 500 μl of serum and incubated at room temperature for 10 min. Then 800 μl of frozen ACN were added, mixed vigorously, and centrifuged at −20 °C (13200 RPM, 10 min). The supernatant was placed in a new tube and partially dried under a nitrogen stream (20 min, 50 °C). To the residue, 100 μl of H_2_O and 600 μl of ethyl acetate were added, mixed and centrifuged (1400 RPM, 6 min). Once the top layer was collected, the LLE process was repeated. The organic extracts were combined, evaporated under a nitrogen stream (50 °C, 15 min) and then derivatized with 100 μl of DAPTAD (200 μg/ml solution in ethyl acetate). A 60-min reaction was conducted at room temperature. In order to stop the reaction, 20 μl of MeOH was added, followed by the evaporation of the mixture under a stream of nitrogen (50 °C, 15 min). After the dissolution of the dry residue in 70 μl of a MeOH/H_2_O mixture (1:1, v:v). 20 μl of sample were injected for LC-MS/MS analysis. The recommended reference range for the method developed by the laboratory is 20–70 pg/mL. Details of the validation process of the two methods are summarized in Table 1.

**Table 1:** Comparison of data from the validation process of two methods: CLIA_LIAISON®XL_ immunoassay and LC-MS/MS techniques.

	CLIA_LIAISON__®__XL_	LC-MS/MS
Limit of detection (LOD)	0,7 pg/ml	10 pg/ml
Lower limit of quantitation (LLOQ)	5 pg/ml	20 pg/ml
Upper limit of quantitation (ULOQ)	5000 pg/ml	200 pg/ml
Accuracy	N/A	98.4 %†
Recovery	94 %	96 %
Repeatability	0.2 %–5.0 %	1.8 %–2.7 %
Reproducibility	3.6 %–6.6 %	1.4 %–8.2 %

Abbreviation: †Average accuracy calculated by comparison of lab result compared to average results reported by other LC-MS/MS labs in DEQAS (Vitamin D External Quality Assessment Scheme program). N/A - not applicable.

### 2.5 Determination of 25(OH)D_3_, 25(OH)D_2_, 24,25(OH)_2_D_3_ and 3-epi-25(OH)D_3_ metabolites by LC-MS/MS technique

A description of the method for determining vitamin D metabolites is included in the supplementary materials.

### 2.6 Statistical analysis

Statistical analysis was performed using R 4.1.2. statistical software (R Core Team (2022) and NCSS version 20.0.8 (NCSS, LLC. Kaysville, Utah, USA)). R: Language and environment for statistical computing by R Foundation for Statistical Computing, Vienna, Austria). Data are presented as n (%) for nominal variables and as the mean with standard deviation (SD) or the median with lower and upper quartiles (Q1; Q3) for continuous variables, depending on the normality of the distribution. Values below the lower detection limit were replaced by half of the detection limit [9]. Verification of normality distribution was based on Shapiro-Wilk test and skewness and kurtosis values. Correlation analysis was based on Spearman's correlation coefficient. Additionally, the comparison between results and the range of agreement between the two analyzed methods were evaluated by the Passing–Bablok regression and Bland–Altman plot. Analysis was based on α = 0.05.

## 3 Results

**Fig. 1:**
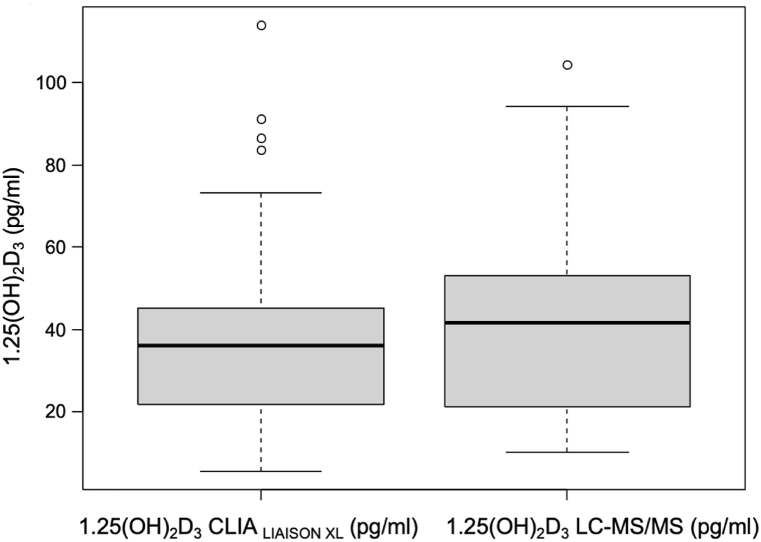
The boxplots display the distribution of 1,25(OH)_2_D results for the CLIA_LIAISON XL_ immunoassay and LC-MS/MS technique. The boxes show the range between the first and third quartiles.

**Fig. 2:**
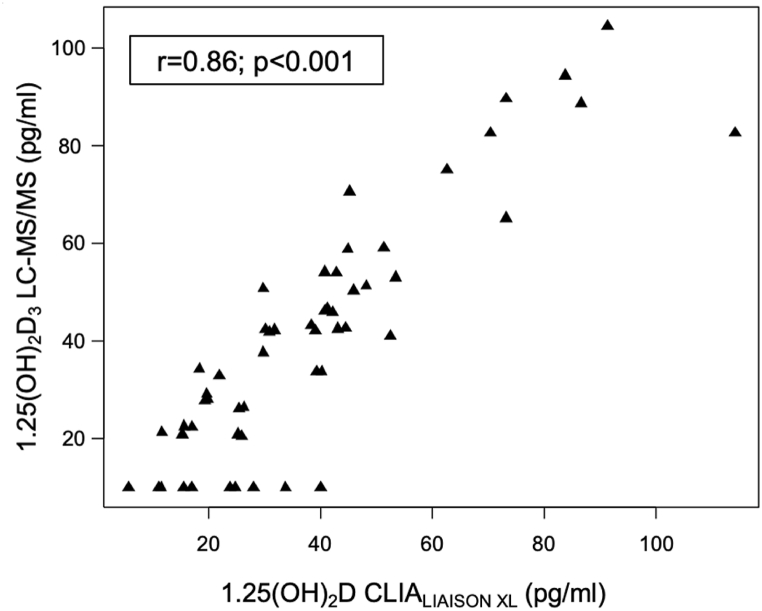
Scatterplot for 1,25(OH)_2_D level determination between CLIA_LIAISON®XL_ immunoassay and LC-MS/MS technique. r, Spearman's correlation.

**Fig. 3:**
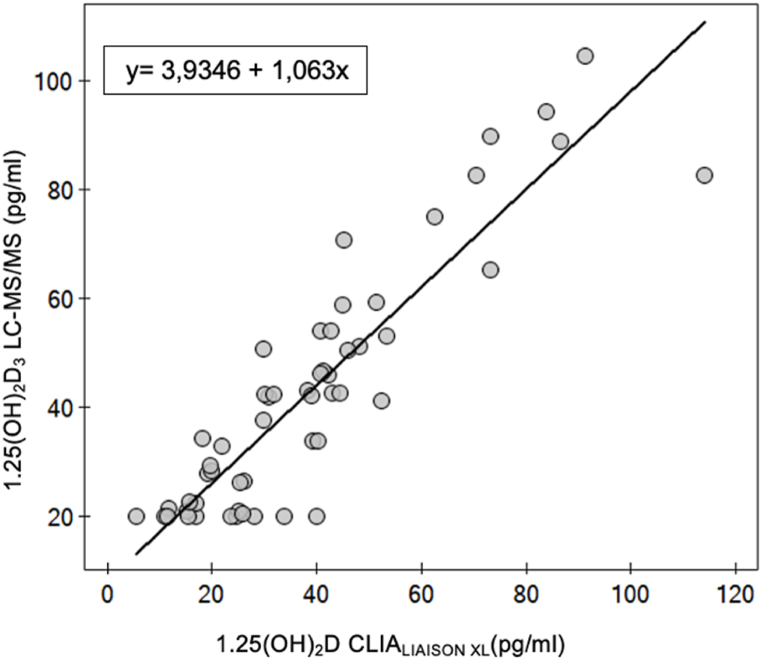
Passing and Bablok regression analyses of CLIA_LIAISON®XL_ immunoassay and LC-MS/MS technique for 1,25(OH)_2_D.

Study population consisted of 54 Caucasian patients, aged from 60 to 96 years old (mean age 73.89 years old), 51.9 % of which were women (n = 28), 48.1 % were men (n = 26), permanently residing in Poland. Characteristics of study group are summarized in Table 2. In the case of the LC-MS/MS technique in 10 out of 54 results of 1,25(OH)_2_D (18.5 %), was below limit of quantification (LOQ) < 20 pg/mL. The CLIA_LIAISON®XL_ immunoassay is characterized by a lower LOD of 0.7 pg/mL but results below 20 pg/mL were obtained by 13 out of 54 (24.1 %). In LC-MS/MS technique, the concentration of 1,25(OH)_2_D was higher compared to the CLIA_LIAISON®XL_ immunoassay (median 41.51 ng/mL (IQR 21.59; 52.61)) vs median 36.0 ng/mL (IQR 22.35; 45.1, p = 0,035) (Fig. 1). Nevertheless, both methods were strongly and positively correlated (r = 0.86; p < 0.001) (Fig. 2). A significant positive correlation was also confirmed for all 25(OH)D_3_ subgroups, based on widely accepted ranges of 25(OH)D_3_ level as shown in Table 3. Moreover, to assess method agreement we conducted a Passing–Bablok regression (Fig. 3). The regression equation revealed an intercept of 3.93 (95 % CI: −25.12–8.31) and a slope of 1.06 (95 % CI: 0.92–1.19) indicating equality and interchangeability of compared methods.

**Table 2:** Characteristics of study group.

	n	Statistics
Total group	54	
Sex, %		
Female	28	51.9
Male	26	48.1
Age (years), mean ± SD	54	73.89 ± 10.08
25(OH)D LIAISON*®XL (ng/ml)*, mean ± SD	53	22.42 ± 13.94
Creatinine (mg/dl), mean ± SD	54	0.80 ± 0.16
Albumin (g/dl), mean ± SD	54	3.59 ± 0.70
PTH (pg/ml), median (Q1; Q3)	54	38.85 (28.33; 47.48)
Calcium (mmol/l), mean ± SD	54	2.23 ± 0.15
Corrected calcium (mmol/l), mean ± SD	54	2.24 ± 0.14
1,25(OH)_2_D CLIA (pg/ml), median (Q1; Q3)	54	36.00 (22.35; 45.10)
mean ± SD		38.34 ± 22.51
1,25(OH)_2_D_3_ LC-MS/MS (pg/ml), median (Q1; Q3)	54	41.51 (21.59; 52.61)
mean ± SD		40.74 ± 24.60
25(OH)D_3_ (ng/ml), mean ± SD	54	20.99 ± 14.28
25(OH)D_2_ (ng/ml), median (Q1; Q3)	54	0.34 (0.19; 0.50)
24,25(OH)_2_D_3_ (ng/ml), median (Q1; Q3)	54	1.24 (0.53; 2.47)
3-epi-25(OH)D_3_ (ng/ml), median (Q1; Q3)	54	0.76 (0.35; 1.27)

Abbreviations: PTH - parathyroid hormone. Corrected calcium = Calcium [mmol/L] + (4 – Albumin [g/dL]) × 0.02.

**Table 3:** Correlation between CLIA_LIAISON®XL_ immunoassay and LC-MS/MS technique for 1,25(OH)_2_D level determination in total study group and in subgroups of patients formed based on the concentration of 25(OH)D_3_.

	n	r	p
Total group	54	0.86	<0.001
25(OH)D_3_ subgroup:(ng/ml)			
<10	12	0.85	0.001
10-20	17	0.93	<0.001
20-30	13	0.77	0.002
>30	12	0.80	0.002

Abbreviation: r – correlation coefficient.

**Fig. 4:**
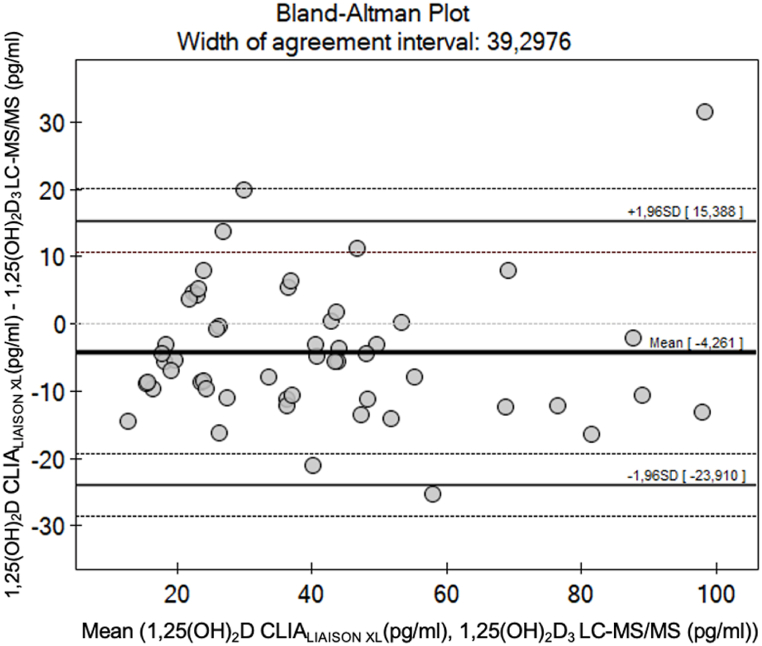
Bland-Altman plot of differences in 1,25(OH)_2_D measured with CLIA_LIAISON®XL_ immunoassay and LC-MS/MS techniques.

In Bland-Altman analysis, the mean bias between the mean results of 1,25(OH)_2_D measured by the CLIA_LIAISON®XL_ immunoassay and the LC-MS/MS technique was −4.26 (1.96 SD, −23.9 to 15.3), indicating that the LC-MS/MS technique gives higher results than the LIAISON®XL (Fig. 4). However, the differences between the results of the two methods (CLIA_LIAISON®XL_ vs. LC-MS/MS) did not change considerably depending on concentration of 1,25(OH)_2_D (Fig. 4).

In the next step we performed correlation analysis between 1,25(OH)_2_D levels obtained with the CLIA_LIAISON®XL_ and LC-MS/MS methods and selected factors (Table 4). The concentration of 1,25(OH)_2_D_3_ was significantly correlated with albumin and calcium levels – this applies to both CLIA_LIAISON®XL_ immunoassay and LC-MS/MS technique. Correlations were comparable for both methods: for albumin r = 0.47; p < 0.001 for CLIA _LIAISON®XL_ and r = 0.46; p < 0.001 for LC-MS/MS, for calcium r = 0.41; p = 0.002 for CLIA _LIAISON®XL_ and r = 0.37; p = 0.006 for LC-MS/MS and for corrected calcium r = 0.40; p = 0.003 for CLIA_LIAISON®XL_ and r = 0.35; p = 0.010 for LC-MS/MS. The remaining parameters (age, creatinine, PTH, 25(OH)D_3_, 24,25(OH)_2_D_3_, 3epi-25(OH)D_3_) were not significantly correlated with 1,25(OH)_2_D_3_ (Table 4).

**Table 4:** Analyses of correlation between 1,25(OH)_2_D results obtained with CLIA _LIAISON®XL_ immunoassay and LC-MS/MS technique and selected biochemical parameters.

	1,25(OH)_2_D_3_ (LIASION® XL) (pg/ml)		1,25(OH)_2_D_3_ (LC-MS/MS) (pg/ml)	
	r	p	r	p
Age (years)	−0.12	0.384	−0.25	0.063
Albumin (g/dl)	0.47	<0.001	0.46	<0.001
Calcium (mmol/l)	0.41	0.002	0.37	0.006
Corrected calcium (mmol/l)	0.40	0.003	0.35	0.010
Creatinine (mg/dl)	−0.01	0.940	−0.05	0.746
PTH (pg/ml)	0.08	0.584	0.11	0.444
25(OH)D_3_ (ng/ml)	0.24	0.082	0.04	0.796
24,25(OH)_2_D_3_ (ng/ml)	0.11	0.443	−0.09	0.509
3-epi-25(OH)D_3_ (ng/ml)	0.22	0.103	−0.002	0.989

Abbreviation: r - Spearman's rank correlation coefficients; Corrected calcium = Calcium [mmol/L] + (4 – Albumin [g/dL]) × 0.02. PTH - parathyroid hormone.

## 4 Discussion

1,25(OH)_2_D is a steroid hormone responsible for intracellular action of vitamin D directed not only at regulating calcium and phosphate homeostasis but also at multiple activities. This bioactive metabolite of vitamin D is a direct ligand for the vitamin D receptor (VDR) which is found in nearly all cells in the human body [10]. Although 1,25(OH)_2_D is not an optimal indicator of vitamin D status, its measurement is relevant in the following clinical situations: substantially increased extrarenal 1α-hydroxylation (in case of sarcoidosis, granulomatous or lymphoproliferative disease), 1α-hydroxylation deficiency (vitamin D dependent rickets type 1, hypophosphatemic rickets, tumor induced osteomalacia (TIO)), vitamin D receptor gene defect (vitamin D dependent rickets type 2) mutation in CYP24A1 gene (cytochrome P450 family 24 subfamily a member 1) causing a disturbance of vitamin D catabolism, in differentiation of hypophosphatemic FGF 23-mediated and non-mediated disorders, PTHrP (parathyroid hormone-related peptide) secreting tumors [5]. However, 1,25(OH)_2_D decreases in chronic kidney disease, its routine evaluation is not recommended [11].

The assessment of 1,25(OH)_2_D began in 1974 with radioreceptor binding assay [12]. Over time, new techniques such as competitive protein binding assays, RIAs (Radioimmunoassays), enzyme immunoassays (EIAs), HPLC (High Performance Liquid Chromatography), GC-MS (Gas chromatography–mass spectrometry) and LC-MS/MS techniques have emerged [3,[13], [14], [15], [16], [17], [18]]. However, the availability of fully automated immunoassays (e.g. CLIA) has made it the dominant method on the market in recent years [15]. Based on The Vitamin D External Quality Assessment Scheme (DEQAS) program data 75 % of the participants used automated immunoassays, among which 59 % were DiaSorin LIAISON®XL, 15 % of the participants used manual immunoassays and 9 % used LC–MS/MS [7].

In the present study the concordance between CLIA and LC-MS/MS for 1,25(OH)_2_D measurement was analyzed. We found that in LC-MS/MS technique, concentration of 1,25 (OH)_2_D was higher compared to the CLIA immunoassay, contrary to previous studies [[19], [20], [21]]. However, a strong positive correlation of 1,25 (OH)_2_D was observed between the two methods. The positive correlation between assays persisted regardless of 25OHD concentration. Furthermore, the regression analysis indicated equality (equal reliability) of the methods.

Despite the fact there is no single gold standard diagnostic method for assessing 1,25(OH)_2_D, both fully automated immunoassay and LC-MS/MS methods have solid scientific and technical basis. Their use in determination of 1,25(OH)_2_D was a breakthrough in laboratory techniques. Automated immunoassay utilizes a highly specific RFP for capture of the 1,25(OH)_2_D and a MAB that is specific to the formed complex of RFP and 1,25(OH)_2_D. Major advantages of the CLIA method include: a small volume of sample, fast result, no need for extraction and purification step, high sensitivity with very low limit of detection (0.7 pg/mL) which may be relevant in cases of very low 1,25(OH)_2_D concentration [22]. In comparison to previously widely used RIA immunoassays, the specificity of the CLIA method is not limited by significant cross-reactivity with other vitamin D metabolites, typically below 0.1 % [[22], [23], [24]]. However, in toxic concentrations of 25(OH)D, the measurement of 1,25(OH)_2_D by the CLIA method may be falsely elevated [25,26]. This phenomenon may be attributed to the interference with 1,24,25(OH)_3_D_3_ or other unidentified metabolites [26]. In our study, the results obtained by CLIA and LC-MS/MS were correlated. It can be attributed to the low to moderate vitamin D concentrations in the study group. Falsely elevated or falsely decreased 1,25(OH)_2_D concentrations may also occur due to cross-reactivity with heterophilic antibodies or human anti-animal antibodies. Another limitation of the CLIA method is limited selectivity and presenting 1,25(OH)_2_D_2_ and 1,25(OH)_2_D_3_ as total 1,25(OH)_2_D. In the study of Tang et al., in pediatric population, the concentrations of 1,25(OH)_2_D determined by the CLIA immunoassay (DiaSorin) were significantly higher than measured by the LC-MS/MS technique which may be explained by the detected 1,25(OH)_2_D_2_ influencing the results [27].

LC-MS/MS is a multistep technique enabling the simultaneous measurement of multiple vitamin D metabolites owing to high analytical selectivity. However, due to 1.000-fold lower concentration in serum, the protein binding, interferences from isomers and isobars, the low ionization efficiency, the measurement of 1,25(OH)_2_D by LC-MS/MS is challenging [28]. LC-MS/MS technique typically uses a complicated 2D chromatography “trap and elute” column system. In the first column analyte is enriched, in the second the separation process occurs [3]. Sample preparation with purification step (protein precipitation, extraction procedure) is crucial for reliable measurement of 1,25(OH)_2_D and separate 1,25(OH)_2_D from other dihydroxylated vitamin D species such as 1β,25(OH)_2_D, 4β,25(OH)_2_D, which have the same molecular weight and may overestimate the result of 1,25(OH)_2_D, especially in patients with very high 25(OH)D [29,30]. Preanalytical immunopurification step is one of the strategies to reduce these interferences [4]. In the presented study, higher results obtained by LC-MS/MS may be partially attributed to lack of additional immunopurification step resulting in false positive signals. Observed differences between various methods are mainly caused by imprecision of one or both methods. Another challenge is insufficient analyte ionization and consequently lower analytical sensitivity, as in recent study, which can be improved by the derivatization of 1,25(OH)_2_D [31]. However, in the presented study, the application of the LC-MS/MS technique with derivatization did not allow to obtain the detection limit similar to CLIA _LIAISON®XL_, not assuring the possibility to determine concentration below 20 pg/mL, which may significantly reduce clinical applications of this method. Various strategies with extra tools and lack of standardization make direct comparison of the LC-MS/MS techniques difficult.

Having considered a greater prevalence of hypercalcaemia in the elderly and demographic ageing we decided to compare the concentration of 1,25(OH)_2_D using CLIA _LIAISON®XL_ and LC-MS/MS methods in geriatric population. In the elderly, acquired unexplained hypercalcaemia or hypophosphatemia are the most common reasons of 1,25(OH)_2_D measurement. Determination of 1,25(OH)_2_D is very useful in diagnosing of PTH-independent hypercalcaemia related with ectopic synthesis of 1,25(OH)_2_D in granulomatous disease like sarcoidosis, tuberculosis, Crohn disease and lymphoproliferative disorders. Although most hereditary vitamin D metabolism disorders are manifested in childhood, loss-of-function mutations in the CYP24A1 gene, leading to inability to inactivate 1,25(OH)_2_D, may cause mild hypercalcaemia and not be diagnosed until adulthood [32]. Moreover, 1,25(OH)_2_D is valuable parameter in distinguishing FGF23-mediated and non-FGF23-mediated hypophosphatemia. In the elderly, the first may be the result of TIO, the second adult-acquired Fanconi syndrome secondary to: multiple myeloma, Wilson disease, primary amyloidosis, light chain nephropathy and heavy metal poisoning (lead, mercury and cadmium) [33,34].

By performing additional analyses, a significant positive correlation between the concentration of 1,25(OH)_2_D obtained by both methods and the albumin and calcium levels were found. 1,25(OH)_2_D like other steroid hormones circulates in the serum bound mainly to vitamin D binding protein (60 %–85 %) and albumin (38 %) [1]. Consequently, changes in serum concentration of these carriers may result in corresponding fluctuations of 1,25(OH)_2_D. The correlations between 1,25(OH)_2_D and calcium level may be explained by the result of 1,25(OH)_2_D activity: stimulating calcium intestinal absorption, renal reabsorption and release from skeletal system.

It needs to be noted that this study is limited by relatively low number of subjects, clinical heterogeneity and lack of patients with hypercalcaemia.

## 5 Conclusions

In our study, we observed high agreement between the CLIA _LIAISON®XL_ immunoassay and the LC-MS/MS technique. Despite wide application of the LC-MS/MS technique in determination of manifold steroid hormones, the results of our study proven that the CLIA _LIAISON®XL_ method may be an optimal choice for elderly patients who often require prompt diagnosis and treatment. Advantages of the CLIA _LIAISON®XL_ method include cost and time efficiency, accessibility and reduced simple sample preparation.

## Funding

This research received no external funding.

## CRediT authorship contribution statement

**Dorota Leszczyńska:** Writing – review & editing, Writing – original draft, Visualization, Resources, Project administration, Methodology, Investigation, Formal analysis, Data curation, Conceptualization. **Alicja Szatko:** Writing – review & editing, Writing – original draft, Methodology, Investigation, Formal analysis, Data curation, Conceptualization. **Magdalena Ostrowska:** Writing – original draft, Investigation, Data curation. **Magdalena Zgliczyńska:** Writing – original draft, Investigation, Data curation. **Konrad Kowalski:** Writing – original draft, Validation, Methodology, Data curation. **Wojciech Zgliczyński:** Writing – review & editing, Validation, Supervision, Funding acquisition. **Piotr Glinicki:** Writing – review & editing, Writing – original draft, Validation, Supervision, Project administration, Methodology, Formal analysis, Conceptualization.

## Declaration of competing interest

The authors declare that they have no known competing financial interests or personal relationships that could have appeared to influence the work reported in this paper.

## Data Availability

Data will be made available on request.
