# Supplementary material for: 1,25-dihydroxyvitamin D in the elderly population: Comparison of liquid chromatography tandem mass spectrometry and CLIA immunoassay (LIAISON®XL) methods
Source: Pract Lab Med. 2025 May 8;45:e00474. doi: 10.1016/j.plabm.2025.e00474 (PMC12158614; doi:10.1016/j.plabm.2025.e00474)
Supplement: Multimedia component 1 [file mmc1.docx]

***2.5 Determination of 25(OH)D_3_, 25(OH)D_2_, 24,25(OH)_2_D_3_ and 3-epi-25(OH)D_3_ metabolites by LC-MS/MS technique***

For the determination of vitamin D metabolites, an isotopic dilution method is used using a high-performance liquid chromatography coupled to tandem mass spectrometry (ID-LC-MS/MS) technique. The main circulating metabolites of vitamin D are analysed including: 25(OH)D_3_, 25(OH)D_2_, epi-25(OH)D_3_ and 24,25(OH)_2_D_3_. A combination of protein precipitation and derivatisation is used to prepare the serum sample (50 µl). Double protein precipitation with acetronitrile is used where isotopically labelled internal standards (^13^C_5_-25(OH)D_3_, ^2^H_6_-24,25(OH)_2_D_3_, ^2^H_3_-25(OH)D_2_ and ^2^H_3_-3-epi-25(OH)D_3_) are added in the first step. Each time the precipitation was carried out for 10 min at room temperature at shaking speeds of 1100 and 3000 rpm, respectively. After centrifugation of the precipitate, the transferred supernatant is evaporated in a stream of nitrogen (12 l/min, 15 min at 55°C). Derivatization using the Diels-Adler reaction is carried out by adding the reagent DAPTAD (4-(4'-dimethylaminophenyl)-1,2,4-triazoline-3,5-dione) in ethyl acetate to the dry residue and the reaction is carried out for 30 min (RT, 450 rpm). After re-evaporation, the dry residue is dissolved in a methanol : water mixture (1:1) and analysed using LC-MS/MS. Chromatographic separation of vitamin D metabolites is carried out using a thermostated column (40°C) in reverse phase mode (Agilent, Zorbax Eclipse XDB-C18, 80Å, 4.6 x 50 mm, 1.8 µm) at a flow rate of 0.8 ml/min in a linear gradient (from 50%:50% to 2%:98%) of water and acetonitrile both with 0.1% formic acid as mobile phases. Analysis is performed on a Shimadzu NexeraXR LC-20AD XR liquid chromatograph using a CTC PALxt autosampler and coupled to a Sciex QTRAP5500 tandem mass spectrometer equipped with an electrospray ion source (TurboV Ion Source). Ions are observed in positive ion mode at 650°C with electrospray generated at 4500 V and nebuliser (GS1) and desiccant (GS2) gas flows of 45 psi and 50 psi respectively. Unique fragmentation reactions are observed in single reaction monitoring (SRM, Q1 mass / Q3 mass) mode for both analytes (25(OH)D_3_ and epi-25(OH)D_3_, 619.5/341.2; 25(OH)D_2_, 631.5/341.2, 24,25(OH)_2_D_3_ 635.5/341.2) and internal standards (^13^C_5_-25(OH)D_3_, 624.5/341.2; ^2^H_3_-epi-25(OH)D_3_, 622.5/344.2; ^2^H_3_-25(OH)D_2_, 634.5/344.2; ^2^H_6_-24,25(OH)_2_D_3_ 641.5/341.2) with ion transmission parameters including declastering potential (DP) and collision energy (CE) of 120eV and 36eV, respectively. Quantitative analysis by isotopic dilution is performed using a 7-point calibration curve prepared by enriching vitamin D free serum (VD-DC Mass Spect Gold®, Gold West Biologicals) with certified reference standards of the vitamin D metabolites. The method presented has been fully validated and is subject to inter-laboratory control within the DEQAS (Vitamin D External Quality Assessment Scheme) programme. The method has satisfactory sensitivity at a limit of quantification (LOQ) of 0.1 ng/mL and appropriate analytical parameters (varying between analytes) including linearity (R2 greater than 0.995) recovery (95%-98%), precision (less than 5%) and accuracy (relative bias less than 10%).
